# Utilisation of small paediatric donor kidneys for transplantation

**DOI:** 10.1007/s00467-018-4073-5

**Published:** 2018-09-20

**Authors:** Samir Damji, Chris J. Callaghan, Ioannis Loukopoulos, Nicos Kessaris, Jelena Stojanovic, Stephen D. Marks, Nizam Mamode

**Affiliations:** 1 Department of Transplantation, Guy’s and St Thomas’ NHS Foundation Trust, London, England SE1 9RT UK; 2 Department of Paediatric Nephrology, Great Ormond Street Hospital for Children NHS Foundation Trust, London, England WC1N 3JH UK

**Keywords:** Kidney transplantation, Paediatric donor, En bloc, Thrombosis, Graft survival, Donation after circulatory death

## Abstract

With the increasing need for kidney transplantation in the paediatric population and changing donor demographics, children without a living donor option will potentially be offered an adult deceased donor transplant of marginal quality. Given the importance of long-term graft survival for paediatric recipients, consideration is now being given to kidneys from small paediatric donors (SPDs). There exist a lack of consensus and a reluctance amongst some centres in transplanting SPDs due to high surgical complication rates, graft loss and concerns regarding low nephron mass and long-term function. The aim of this review is to examine and present the evidence base regarding the transplantation of these organs. The literature in both the paediatric and adult renal transplant fields, as well as recent relevant conference proceedings, is reviewed. We discuss the surgical techniques, long-term graft function and rates of complications following transplantation of SPDs. We compare graft survival of SPDs to adult deceased donors and consider the use of small paediatric donors after circulatory death (DCD) organs. In conclusion, evidence is presented that may refute historically held paradigms regarding the transplantation of SPDs in paediatric recipients, thereby potentially allowing significant expansion of the donor pool.

## Introduction

In many countries, rates of living donor renal transplantation appear to have reached a plateau or have even started to fall. There were approximately 1050 living kidney donors each year from 2010 to 2017 in the UK, whilst in the USA there has been a steady decline from 6279 living kidney donors in 2010 to 5627 donors in 2016 (Table 1) [1, 2]. At the same time, in the UK, the proportion of adult organs from donors after circulatory death (DCD) has dramatically increased, and now accounts for 40% of all deceased donors [1]. With adult donor demographics changing, the quality of organs from donors following brain death (DBD) is worsening; donors are typically older, have higher BMIs and possess co-morbidities such as hypertension and diabetes. In the UK, the number of deceased donors over the age of 60 years has risen from 17% in 2007 to 36% in 2016, whilst the number of donors over the age of 70 years has risen from 3 to 14% over the same period. Similarly, the proportion of hypertensive deceased donors rose from 18% in 2003 to 31% in 2012 [3]. The resulting decline in the quality of donor organs has meant that by 2012 only 1 in 5 of all deceased donors were considered as ‘ideal’, i.e. aged under 60 years, with a body mass index (BMI) under 30 kg/m^2^ and no history of smoking or hypertension. The implication of changing donor demographics is that many children in the UK and USA that do not have a living donor option will be offered an adult deceased donor transplant potentially of marginal quality, despite efforts to match with organs possessing the lowest Kidney Donor Profile Index (KDPI). Whilst paediatric Eurotransplant kidneys are prioritised for paediatric recipients, this has not historically been the case in the UK or the USA due to concerns regarding technical complications. However, given the importance of long-term graft survival for paediatric recipients and recent paradigm shifts within individual units, consideration is now being given to new sources of organs which might sustain excellent renal function with longer graft survival. Attention has therefore turned to the use of organs from small paediatric donors (SPD).

**Table 1:** Donor type over the last 8 years in the UK and USA

	2010	2011	2012	2013	2014	2015	2016	2017
Living donors, USA ^1^	6279	5773	5619	5735	5538	5631	5627	5817
Living donors, UK ^2^	1062	1046	1055	1101	1148	1092	1078	1043
Total deceased donors, USA ^1^	7241	7434	7421	7548	7763	8250	9116	9401
Total deceased donors, UK ^2^	959	1010	1088	1212	1320	1282	1364	1413
DCD donors, USA ^1^	943	1057	1107	1207	1292	1494	1684	1883
DCD donors, UK ^2^	335	373	436	507	540	510	579	584
Deceased donors under 17 years, USA ^1^	956	881	852	873	842	939	934	896
Deceased donors under 17 years, UK ^2^	38	40	44	36	52	51	55	57

*DCD* donors after circulatory death

^1^US annual figures are for calendar years; 1 Jan 2016 to 31 Dec 2016 is given as 2016

^2^UK yearly figures are for financial years; 2009/2010 is given as 2010

The proportion of paediatric transplants in the USA utilising organs from deceased donors under 10 years old has declined dramatically from 35% in 1987 to 5% in 2010 [4]. This is only partially accounted for by the increase in live donation during this time period. A UK registry review of the use of paediatric donors between 1997 and 2011 identified 17 renal transplants from donors under the age of 2 years old, with 5 donors under the age of 1 year and no neonatal donors. Of these organs, 82% were transplanted en bloc into adult recipients [5]. En bloc refers to retrieving both kidneys and major blood vessels together, with subsequent anastomoses of the donor aorta and inferior cava to the recipient vessels [6]. Historically, high vascular and urologic complication rates had made utilisation of these grafts challenging in both paediatric and adult recipients.

There exist a lack of consensus and a reluctance amongst centres in transplanting SPD organs due to an increased risk of thrombosis of the donor vessels. For the purposes of this review, we have defined a SPD as a donor weighing less than 20 kg, though definitions vary in the literature. An average child of 20 kg is approximately 6 years old, with a renal length of approximately 8 cm. A univariate analysis by Singh et al. of NAPRTCS registry data showed significantly higher rates of SPD graft loss due to thrombosis in paediatric recipients under 2 years old compared to those over 12 years old (9.0 vs 3.5%; *p* = 0.01). Furthermore, there was a graft thrombosis rate of 8.3% (*n* = 386) in organs from donors under the age of 5 years, compared to 3.2% (*n* = 1667) in donors over 10 years [7].

There are concerns that the small nephron mass provided by small kidneys from SPDs may not provide adequate renal function in adult recipients with a potential risk of hyperfiltration-associated renal injury and graft loss. However, Al-Bader et al.’s retrospective review of paediatric small kidneys transplanted into adult recipients, when compared to matched adult donors, showed no long-term deterioration in graft function and demonstrated similar creatinine clearances in both groups at various end points [8].

Due to these concerns, the use of SPD organs in both adult and paediatric transplantation remains controversial and therefore raises questions that should be addressed. What is the long-term graft function and survival when compared to adult donor organs? What is the additional risk associated with the use of SPD organs and how does this compare to organs from elderly deceased donors? Should small kidneys be used exclusively for paediatric recipients? Should they be implanted as single kidneys or en bloc? Is there a minimum weight for the donor? Can we transplant organs from small paediatric DCDs? Should a paediatric transplant surgeon be present at the time of retrieval to facilitate organ assessment? If so, is the extra resource allocation cost and time effective, when the return is relatively low?

This review article aims to address these points and to determine if the use of SPD organs are appropriate and under what circumstances.

## Graft survival

Studies examining outcomes following transplantation using SPDs (with more than 20 recipients) are shown in Table 2 [5, 11–15, 18, 20, 22, 23, 25–28, 30, 31]. In one of the largest studies to date, a US-based registry analysis of 2449 renal transplants from paediatric donors under 20 kg [11], the authors demonstrated a 5-year graft survival rate of 55% for single-organ kidney transplants and 73% for en bloc kidney (EBK) transplants, with a hazard ratio of 0.96 per 1 kg increase in donor weight for graft loss. These results are comparable to those of a large UNOS registry review, which identified a mean 5-year graft survival rate of 56% in 2198 transplants where the donor’s age was 5 years old or less (*p* < 0.01) [12]. This was compared to a 65% graft survival rate in 7767 transplants where the donor’s age was between 12 and 17 years old. The authors also identified a 10% graft thrombosis rate in kidneys from donors aged 5 years or under, with an overall 1-year graft survival of 82% with any paediatric donor, compared with 84% with adult donor organs (*p* < 0.01). In a European registry analysis of 429 transplants from paediatric donors aged between 0 and 5 years into paediatric recipients, the authors sought to examine the association of donor-recipient age combination with 5-year graft survival [14]. Performing sub-group analyses for recipient age ranges (0–3 years, 0–5 years, 6–11 years and 12–19 years), they demonstrated 5-year graft survival rates of 70%, 75%, 81% and 83% respectively.

**Table 2:** Outcomes after transplantation from SPD kidneys: ranked by *n = x*

Author	Date range	*n = x*	Definition of SPD	Outcome – graft survival	Comments
Dharnidharka [9]	1987–2003	3957	0–5 years	1-year graft survival	EBK GT rate 4% SOK GT rate 3%
				▪ SOK – 81% ▪ EBK – 85%	
				3-year graft survival	
				▪ SOK – 68% ▪ EBK – 76%	
				5-year graft survival	
				▪ SOK – 63% ▪ EBK – 71%	
Bhayana [10]	1998–2006	3198	0–5 years	1-year graft survival	EBK GT rate 5% SOK GT rate 3%
				▪ SOK – 81% ▪ EBK – 85% ▪ Adult SCD – 90% ▪ Adult ECD – 83%	
				10-year graft survival	
				▪ SOK – 53% ▪ EBK – 64% ▪ Adult SCD – 57% ▪ Adult ECD – 40%	
Pelletier [11]	1993–2002	2449	< 20 kg	5-year graft survival	HR 0.96 for GL per 1 kg increase in donor weight
				▪ SOK – 55% ▪ EBK – 73%	
Bresnahan [12]	1988–1995	2198	0–5 years	1-year graft survival	SOK + EBK GT rate 10%
				▪ SOK + EBK – 74%	
				5-year graft survival	
				▪ SOK + EBK – 56%	
Maluf [13]	2005–2010	1531	< 20 kg	1-year graft survival	1-year transplant outcomes by kg weight strata
				▪ SOK – 69% (8 Kg) ▪ EBK – 80% (8 Kg) ▪ SOK – 86% (20 Kg) ▪ EBK – 91% (20 Kg)	
Chesnaye [14]	1990–2013	429	0–5 years	5-year graft survival	Association of donor-recipient age combination on outcomes
				▪ 70% (recipient age 0–3 years) ▪ 75% (recipient age 0–5 years) ▪ 81% (recipient age 6–11 years) ▪ 83% (recipient age 12–19 years)	
Yaffe [15]	1996–2013	167	10–20 kg	1-year graft survival	All into paediatric recipients
				▪ SOK – 90% ▪ EBK – 86%	
				5-year graft survival	
				▪ SOK – 61% ▪ EBK – 73%	
Winnicki [16]	2000–2013	126	Not defined	1-year graft survival	All into paediatric recipients
				▪ EBK – 86% ▪ Adult SCD – 93%	
				5-year graft survival	
				▪ EBK – 64% ▪ Adult SCD – 69%	
Diaz [17]	1990–2012	100	Not defined	12-year graft survival	GT rate 15%
				▪ EBK – 79% ▪ Adult SCD – 69%	
Satterthwaite [18]	1984–1995	91	1–4 years	1-year graft survival	
				▪ SOK – 64% ▪ EBK – 82%	
Thomusch [19]	1989–2008	78	Not defined	1-year graft survival	
				▪ EBK – 83% ▪ Adult SCD – 90%	
				5-year graft survival	
				▪ EBK – 76% ▪ Adult SCD – 78%	
				10-year graft survival	
				▪ EBK – 74% ▪ Adult SCD – 56%	
Sureshkumar [20]	1990–2001	72	Not defined	1-year graft survival	GT rate 13% - Mean age 17 months
				▪ EBK – 82% ▪ Adult LD – 93%	
Gander [21]	2000–2015	60	0–6 years	1-year graft survival	
				▪ All SPD organs – 1 year. GS 81%	
				5-year graft survival	
				▪ All SPD organs – 5 years. GS 70%	
Strey [22]	1992–1999	56	0.50.5–48 months	1-year graft survival	GT rate 21% - donors < 1 years age (heparinisation used)
				▪ EBK – 78% ▪ Adult DD – 92%	
Sharma [23]	2000–2011	52	< 10 years old	5-year graft survival	All into adult recipients
				▪ SOK – 81% ▪ EBK – 94% ▪ Adult SCD – 75% ▪ Adult ECD – 75%	
Borboroglu [24]	1994–2001	48	0–2 years	2-year graft survival	
				▪ SOK – 93% ▪ EBK – 77%	
Dave [5]	1997–2011	47	< 2 years	1-year graft survival	No difference compared to older paediatric donors. Median 9 years. PS 100%
				▪ SOK + EBK – 83%	
Bretan [25]	1993–1996	40	0–5 years	1-year graft survival	GT rate 2.5%
				▪ EBK – 100%	
				2-year graft survival	
				▪ EBK – 85%	
Sui [26]	2012–2014	38	< 15 kg	1-year graft survival	All into paediatric recipients
				▪ All SPD organs – 89% ▪ SOK – 96% ▪ EBK – 70%	
Basiri [27]	2006–2013	36	Not defined	1-year graft survival	GT rate 5.5%
				▪ EBK + SOK – 90% ▪ Adult DD – 92%	
Hobart [28]	1990–1997	33	< 4 years	1-year graft survival	GT rate 15%
				▪ EBK – 87% ▪ Adult DD – 84%	
Mohanka [29]	2002–2006	33	< 15 kg	1-year graft survival	All into adult recipients
				▪ SOK – 86% ▪ EBK – 79%	
Salvatierra [30]	1969–1973	32	1–9 years	6-month graft survival	
				▪ SOK – 50%	
Zafarghandi [31]	2004–2009	23	< 16 years	1-year graft survival	All into adult recipients
				▪ EBK + SOK – 96% ▪ Adult DD – 91%	
				5-year graft survival	
				▪ EBK + SOK – 85% ▪ Adult DD – 85%	

*GS* graft survival, *GT* graft thrombosis, *HR* hazard ratio, *GL* graft loss, *LD* live donor, *DD* deceased donor, *SOK* single organ kidney, *EBK* en bloc kidney, *PS* patient survival

These reports consistently demonstrate good long-term graft survival but with an increase in the risk of early graft loss from thrombosis. However, it is important to consider that a majority of these analyses are historical and there exist no recent large-scale reviews or randomised controlled trials. Furthermore, the quality of deceased donor organs and the use of anti-thrombotic protocols have evolved since this data was collected.

A number of investigators have sought to compare the outcomes of SPD kidney transplants to matched cohorts of adult standard criteria donor (SCD) transplants [17, 22, 23, 28]. In a large retrospective review of a single centre experience, 100 EBK transplants were compared to adult matched DBD transplants (mean age 1.8 years vs 42.9 years respectively). Graft survival at 1 year was slightly inferior in the EBK group (82 vs 88.9% respectively) with a graft thrombosis rate of 15% in the former [17]. The authors demonstrated better graft survival in the EBK group compared to the adult group at 12-year follow-up (78.7 vs 69.2% respectively). Similarly, Sharma et al. in a retrospective single-centre review compared outcomes of both EBK and single kidney transplants to an adult cohort of SCD and extended criteria donor (ECD) transplants [23]. Graft survival at 5 years was significantly better within the EBK and single kidney groups (94% and 81% respectively, *p* = 0.02) compared to the adult donor groups, with both SCD and ECD 5-year graft survival at 75%. However, a small review by Stey et al. demonstrated contrasting findings, with inferior outcomes in the SPD group [22]. The authors in a review from 1992 to 1999 of 56 EBK transplants compared outcomes to adult deceased donor transplants in the same centre. One-year graft survival was inferior at 78% compared to 92% in the adult cohort. However, it is important to note that the graft thrombosis rate was unusually high (21%) despite the routine use of heparinisation, which may allude to technical difficulties when carrying out these challenging procedures.

Despite the historically high technical failure rate associated with transplantation of SPDs, it appears overall that long-term outcomes are superior when compared to matched cohorts of adult SCD grafts. The published outcomes are excellent even when compared with outcomes following living donor transplantation [20, 23]. Clearly, more data is needed; however, perhaps the better comparison is with the long-term outcomes following ECD organ transplantation. There is a paucity of data available, with only one large UNOS registry review comparing graft survival of SPDs and ECDs [10]. Bhayana et al. analysed 3198 SPD transplants, comprising of both EBKs and single kidney transplants. Graft survival at 1 year was 85% and 81% respectively and was comparable to ECD 1-year graft survival of 83%. Graft survival at 10 years was significantly better in the EBK and single kidney transplant groups compare to that in the ECD groups (64% + 53% vs 40%). These findings confirm the role of SPD organs for transplantation with measurably excellent long-term results; however, the next consideration is whether EBK transplantation might result in better outcomes by increasing nephron mass and fewer complications by minimising vascular thrombosis [24].

## EBK versus single organ kidney transplantation

Unsurprisingly, there are currently no randomised controlled trials comparing outcomes following EBK transplantation to single kidney transplantation, in part due to the low frequency of such transplants at centres throughout the world. Comparison of the techniques is confounded by the fact that kidneys undergoing en bloc transplantation are typically smaller with a lower mass than those that may be considered for single implantation. Furthermore, damage to the en bloc specimen at retrieval may result in one organ being discarded and the other organ being transplanted singly. This will likely increase the risk of complications, reduce nephron mass and affect long-term survival.

Studies which have attempted to compare EBK and single kidney transplantation are shown in Table 2. Several small, single-centre reviews have shown overall excellent outcomes following both techniques; however, they demonstrate conflicting results as to which technique is superior [18, 23, 24, 26, 29].

The strongest evidence that EBK transplantation might provide superior graft survival comes from several large US registry reviews [9–11, 15]. In the largest published review of UNOS registry data available to date, Dharnidharka et al. sought to examine the outcomes following single kidney and EBK transplantation in 3957 recipients [9]. Graft survival at 1, 3 and 5 years in the single kidney cohort were 81%, 68% and 63% respectively whilst graft survival in the EBK cohort was 85%, 76% and 71% respectively. EBK transplantation demonstrated significantly better graft survival at the various follow-up time points (*p* < 0.001). The previously described registry review by Bhayana et al. in 3198 SPD organ recipients demonstrated superior EBK transplantation outcomes at 1 and 10 years (85% and 64%) compared to single kidney transplantation (81% and 53%) at similar time points [10]. Despite the increased risk of early graft thrombosis for EBK and single kidney transplantation (5% and 3% respectively, compared to 1.8% for SCD and 2% for ECD transplantation), SPDs demonstrated excellent outcomes, with EBK transplantation associated with the best long-term graft and patient survival. Similarly, Pelletier et al*.* analysed 2449 recipients of SPD kidneys [11]. Graft survival for the EBK transplant groups was superior than that of single kidney transplantation (73% vs 55%), with a hazard ratio of 0.96 for every kilogramme increase in donor weight. Satterthwaite et al. in a single-centre review examined the use of kidneys from very young deceased donors (under 4 years old), demonstrating better graft survival following EBK transplantation, with 1- and 5-year graft survival of 82% and 70% respectively [18]. Recipients undergoing single kidney transplantation exhibited 1- and 5-year graft survival of 64% and 40%. However, the authors noted that kidneys from donors less than 2 years had poorer long-term outcomes, irrespective of the technique used. Mitrou et al. have also examined outcomes following EBK transplantation of very small kidneys from infant donors (body weight < 10 kg) [32]. The authors identified 28 EBK transplant recipients, of which 11 were from donors weighing less than 10 kg. Serum creatinine at 1, 3 and 5 years was similar between small and large donors (donor weighing > 10 kg) groups. Interestingly, both groups exhibited marked volumetric growth within the first year of follow-up. At the time of transplantation, grafts from the smaller donors were significantly smaller (28 ± 9 mm^3^ vs 45 ± 12 mm^3^, *p* < 0.01); however, at 1-year post-transplantation, the graft sizes from both groups were similar (88 ± 44 mm^3^ vs 93 ± 52 mm^3^, *p* = NS).

Whilst these results are encouraging, there still exist a paucity of strong evidence of the long-term outcomes and the feasibility of utilising kidneys from infant donors. Several investigators have suggested that there may be a lower age threshold, beyond which results are inferior [25, 33]. However, much of this commentary is based on historical data and further analysis of contemporary practice and outcomes is needed.

There is compelling evidence to suggest that overall EBK transplantation provides excellent graft survival and function. However, to maximise the utilisation of a scarce resource and extend access to the benefits of transplantation to as many recipients as possible, the question remains as to whether single kidney transplantation to two recipients is more appropriate. Laurence et al. constructed a decision analysis model to predict the outcome in life years for patients on the transplant waiting list depending on whether they received an EBK or single kidney transplant [34]. The authors identified a greater net gain in overall life years performing single kidney transplantation as the technique yields two recipients per donor, which compensated for the reduced long-term graft survival and function. The investigators also confirmed that single kidney transplant technique resulted in a net loss of life years for donors weighing less than 10 kg. At this threshold, the inferior outcomes from single kidney transplantation are not compensated for by the generation of twice the number of recipients. Whilst the inference may be that EBK transplantation is the optimal technique for very small kidneys from donors weighing less than 10 kg, the study was not able to draw firm conclusions to confirm this.

## Use of SPD organs in paediatric recipients

To expand the donor pool and decrease waiting times, there has been increased utilisation of SPD organs transplanted into adult recipients, with the very small organs transplanted en bloc. Although an increased risk of graft thrombosis and hyperfiltration injury has been demonstrated with the use of EBK transplants, reports in adult recipients show excellent outcomes. Currently, only a small percentage of renal transplantation in paediatric recipients utilise SPDs. Children undergoing renal transplantation pre-emptively or after a short waiting time have not only survival benefit but also better growth and cognitive development compared with children receiving dialysis. Therefore, expanding the donor pool in the paediatric population is of paramount importance to enable transplantation as soon as possible in this cohort.

The large US-based registry reviews previously described analyse long-term outcomes in combined adult and paediatric recipient groups; none however perform sub-group analysis to assess the outcomes of using SPD kidneys in paediatric recipients [9–12]. Studies analysing long-term outcomes of SPD kidney transplants in paediatric recipients are largely single-centre and low-volume analyses; however, several recent larger studies have demonstrated promising results. Winnicki et al., using UNOS-derived data, assessed the outcomes of 126 paediatric recipients that had undergone EBK transplantation from SPDs, comparing to paediatric recipients that had undergone transplantation from adult SCDs [16]. Graft survival in the EBK cohort was 64% at 5 years, with a hazard ratio of 1.15, whilst adult SCD transplantation demonstrated a 5-year graft survival of 69%. In a recent US-based registry review, Yaffe et al. identified 167 children that had undergone transplantation from SPD organs (both EBK and single kidney transplantation) and 2350 children transplanted with adult SCD organs [15]. Paediatric recipients of adult SCD kidneys had the highest 1-year graft survival (94%), with marginally inferior outcomes noted in the EBK and single kidney cohorts (86% and 90% respectively). The investigators noted that 5-year graft survival of adult SCD and EBK transplantation (72.6% vs 72.8% respectively) was comparably better than single kidney transplantation (61%). In the study of Filler et al*.*, outcomes of paediatric renal transplant recipients from donors less than 6 years old were reported and compared to those from older donors. Graft survival was comparable in both groups at 1 year (77 vs 76%) and 5 years (55 vs 60%) [35].

Despite these promising results, there is evidence that suggests allograft function and survival are poorer when both recipient and donor are young. Singh et al. in a univariate analysis of 4394 transplants, showed that graft loss due to thrombosis was significantly higher in children less than 2 years of age compared to that in older groups (9% vs 3.5% when recipients are older than 12) [7]. Recipients of kidneys from donors less than 5 years old had a significantly higher thrombosis rate than older donor groups (8.3% vs 3.2% in donors greater than 10 years old). Bresnahan et al. demonstrated a reduced graft survival in paediatric transplant recipients with SPD organs where the donor was less than 5 years old, due to a high rate of complications [12]. However, graft survival from older paediatric donors was comparable to that of adult donors.

It appears that the risk of transplantation with very small SPD kidneys in both children and adult recipients is greatest in the early postoperative period. However, it is clear that children receiving organs from older paediatric donors have comparable or superior long-term functional outcomes compared to those that receive adult allografts.

## Use of paediatric DCD organs

The use of DCD organs in adults has become an accepted way of recovering more organs for transplantation and has allowed expansion of the donor pool. However, increasing acceptance and utilisation of paediatric DCD organs remain challenging for a variety of reasons. There exist limited experience and comfort with transplanting such organs and few standardised protocols for their use, due to concerns over compromised graft survival. There is also considerable ethical and emotional concerns surrounding the use of such organs.

Workman et al.’s recent review of data from the Organ Procurement and Transplant Network (2001–2010) showed a 10% decrease in the use of paediatric deceased donor solid organs over the 10-year period [36]. During this period, only 42 paediatric DCD kidneys were transplanted into paediatric recipients, whilst 805 paediatric DCD kidneys were transplanted into adult recipients. The investigators make no distinction between size of the organ or age of donor and therefore no comment can be made on the use of SPD organs. Vries et al., in a Dutch multi-centre retrospective review, identified 91 adult recipients that were transplanted with paediatric DCD kidneys, where outcomes were compared to a larger (*n* = 405) cohort of paediatric DBD organ recipients [37]. The authors showed the DCD cohort to have a higher primary nonfunction rate (9% vs 2% respectively) and higher delayed graft function (DGF) rate (48% vs 8% respectively) than the DBD cohort. Estimated GFR at 1 year was comparable between the DCD and DBD groups (57 mL/min vs 58 mL/min, respectively) and similar at 5 years (62 mL/min vs 57 mL/min, respectively) in those with functioning grafts.

In contrast to similar studies examining DCD paediatric transplantation, Vries et al. identified that 12% of the DCD kidneys within their analysis were considered Maastricht category 2, defined as uncontrolled donation following unsuccessful resuscitation [37]. The authors did not however include a sub-group analysis to differentiate the outcomes between uncontrolled and controlled donations. Uncontrolled donation refers to donors who suffer an unexpected cardiac arrest and are either brought into hospital dead (Maastricht category 1) or when death is declared in hospital following unsuccessful attempts at cardiopulmonary resuscitation (Maastricht category 2).

Dion et al. in a recent single-centre analysis identified 21 adult recipients of EBK transplants from paediatric donors under the age of 4 years old, of which four organ pairs were obtained from DCD donors [38]. DGF ensued in 2 of 4 DCD organ recipients and 3 of 17 DBD organs. Graft function at 1 year was comparable with GFR of 80.7 mL/min in the DCD cohort and 85.7 mL/min in the DBD cohort. No grafts were lost due to thrombosis during the follow-up period. Abt et al. reviewed the UNOS database and published a review of 4026 renal transplants in paediatric recipients [39]. They identified 26 (0.6%) transplants that used DCD donors. Of the DCD donors, 10 were from paediatric donors and the remaining from adult donors. The authors demonstrated graft survival at 1 and 5 years in the entire DCD group (83% and 74% respectively) and in the DBD group (90% and 74% respectively). However, the investigators did not perform sub-group analyses on the DCD group, to compare paediatric versus adult donors.

At present, therefore, there is minimal data regarding the use of small paediatric DCD organs in children. Whilst results obtained from adult DCD organs, when implanted into adults, are equivalent to those from DBD organs, a recent UK registry data analysis has demonstrated that adult DCD organs, when transplanted into children, possess outcomes that are comparable to DBD organs or organs from live donors [40]. However, the higher rates of delayed graft function, the potential impact of additional warm ischaemia on rejection rates and the increased risk of early graft thrombosis in small kidneys means that the use of DCD organs from SPDs should be done with care, until adequate data is available. To gain a wider acceptance of the use of small paediatric DCD kidneys for transplantation, there is a need for greater assessment of long-term graft function and survival, and this needs to be optimised for younger recipients.

## Discussion

Kidney transplantation is the treatment of choice for children with end stage renal disease. It is well established that patient survival, quality of life, growth and cognitive development are superior with transplantation compared to dialysis [41]. As donor demographics change, with poorer quality deceased donor kidneys on offer and rates of live donation falling, additional stratagems are being sought to expand the donor pool. The use of small paediatric donor kidneys is now being considered for both adult and paediatric recipients. Efforts to reduce waiting times in the adult population have resulted in increased acceptance of the use of SPD kidneys in adult recipients, such that greater than half of all major US transplant centres are actively performing either EBK or single kidney transplants [13]. A number of large national registry reviews have shown excellent outcomes with both EBK and single kidney transplantation in adult recipients, with graft survival and function either comparable or superior to those of SCD and ECD transplantation. Currently, only a small percentage of EBK transplants are performed in paediatric recipients, and as a result there are limited studies demonstrating long-term outcomes. Several small and single-centre reviews have been published, which demonstrate promising results; furthermore, two recent large US-based registry reviews have examined long-term outcomes after transplantation with SPD organs in children. Yaffe et al*.* and Winnicki et al. both demonstrated marginally inferior outcomes in SPD organs at 1-year follow-up compared to adult SCD organs; however, this had equalised by follow-up at 5 years with comparable survival rates between organ types and techniques [15, 16]. Winnicki et al. also showed that after adjusting for co-variates, the hazard ratio for graft failure of EBK transplantation was similar to that of SCD transplantation 1.04 (95% CI, 0.71–1.51; *p* = 0.85), whilst the EBK transplantation group had significantly shorter wait times, between activation and transplantation, compared to that of the SCD group (157 days vs 208 days, *p* = 0.03).

Historically, there has been reluctance to transplant SPD organs in children, due to concerns of a higher incidence in surgical complications; however, the more recent published reviews have shown lower complication rates [5, 16]. This is likely a result of increased experience in EBK transplantation, shorter cold ischaemia times, increasing use of antithymocyte globulin at induction, technical refinements and the combined post-operative use of heparin and antiplatelet agents. These factors suggest that EBK transplantation is a safe and feasible option, offering superior graft and patient survival characteristics in children. Further investigation is required to assess longer outcome (> 10 years) of EBK and single kidney transplantation in children, along with assessing the risk of hyperfiltration injury and the feasibility of the potential use of DCD kidneys.

National and locally led strategies are needed to reduce paediatric transplant waiting times and increase the deceased donor pool by greater utilisation of SPD organs. Improved utilisation requires tackling several weaknesses in the organ transplant pathway. SPD organ procurement rates are low and whilst potential causal agents have been considered, specific rationales have not been analysed and documented [11, 36, 42]. This needs greater consideration with strategic implementation at a national organisational level (i.e. UNOS, NHSBT). Secondly, many retrieved kidneys are discarded at either retrieval or recipient site. Concerns regarding organ function and potential mismatches, donor medical history and recipient-related issues tend to result in declined organs in a small number of cases. However, the dominant concerns centre around organ anatomy and damage at time of retrieval. Retrieval surgeons should be appropriately trained and experienced to ensure that SPD organs are safely recovered. Short cold ischaemia times and meticulous back table preparation are of paramount importance.

Another opportunity to increase transplantation rates would be to lower the threshold for en bloc splitting, to allow single kidney transplantation into two different recipients. Further evidence is required to determine the optimal donor weight for single-organ kidney and EBK transplantation; however, we would suggest a sensible minimum threshold of 15 kg when considering single kidney transplantation from SPDs. The decision to transplant organs from SPDs and the choice of performing EBK versus single kidney transplantation require careful consideration and assessment to ascertain that maximal benefit is attained for individual recipients and the remaining patients on the waiting list. When considering splitting, the surgeon should also consider the surgical approach and potential space required in the recipient. EBK transplantation into adult recipients within the extraperitoneal iliac fossa is well recognised and commonly performed. A similar approach may also be suitable in older children and adolescents; however, smaller children are likely to need an intra-peritoneal approach. In all but the smallest children, there is adequate space to transplant small EBKs within the abdominal cavity without developing abdominal compartment syndrome. However, there are no models described within the literatures to determine the available space or to stratify the risk of compartment syndrome; the authors would therefore recommend a case by case assessment by an experienced surgeon.

There is no doubt that centre volume and surgeon experience will affect graft survival rates, especially in very small paediatric recipients. Centralising specialist services in high-volume centres will result in better patient and graft outcomes. In the UK, two NHS hospital trusts (Guy’s Hospital and Leeds Hospital) have been appointed as national specialist leads for utilisation of organs from donors below 2 years old. Discussions are ongoing in the UK about the provision of expertise for such retrievals. Clearly, evidence will be required that any additional resources result in a significant benefit.

## Conclusion

In conclusion, many SPD kidneys are excellent quality organs and have the potential to significantly expand the paediatric donor pool. These organs will allow the benefits of transplantation to be extended to many children and provide good long-term graft function, but carry an increased risk of perioperative thrombosis. It is likely that en bloc organs are to be preferred for small donors, although the cut-off point is unclear. Similarly, the minimum donor weight is uncertain, although organs from neonates have been transplanted. Using organs from small paediatric donors for implantation into children is feasible, and probably offers at least equivalent outcomes to ECD donors, but organs from very small donors should probably not be used widely until further data is available. We advocate a careful but considered approach to the use of these organs. We encourage stratagems to increase retrieval and utilisation rates as well as techniques to improve long term-outcomes from transplantation of SPD organs.
